# Vernalization and the chilling requirement to exit bud dormancy: shared or separate regulation?

**DOI:** 10.3389/fpls.2014.00732

**Published:** 2014-12-17

**Authors:** Amy M. Brunner, Luke M. Evans, Chuan-Yu Hsu, Xiaoyan Sheng

**Affiliations:** ^1^Department of Forest Resources and Environmental Conservation, Virginia Polytechnic Institute and State UniversityBlacksburg, VA, USA; ^2^Department of Biology, West Virginia UniversityMorgantown, WV, USA; ^3^Institute for Genomics, Biocomputing and Biotechnology, Mississippi State UniversityStarkville, MS, USA

**Keywords:** dormancy, vernalization, adaptation, *FT*, *TFL1*

## Abstract

Similarities have long been recognized between vernalization, the prolonged exposure to cold temperatures that promotes the floral transition in many plants, and the chilling requirement to release bud dormancy in woody plants of temperate climates. In both cases the extended chilling period occurring during winter is used to coordinate developmental events to the appropriate seasonal time. However, whether or not these processes share common regulatory components and molecular mechanisms remain largely unknown. Both gene function and association genetics studies in *Populus* are beginning to answer this question. In *Populus*, studies have revealed that orthologs of the antagonistic flowering time genes *FT* and *CEN/TFL1* might have central roles in both processes. We review *Populus* seasonal shoot development related to dormancy release and the floral transition and evidence for *FT/TFL1-*mediated regulation of these processes to consider the question of regulatory overlap. In addition, we discuss the potential for and challenges to integrating functional and population genomics studies to uncover the regulatory mechanisms underpinning these processes in woody plant systems.

## INTRODUCTION

Variation in responses to prolonged periods of cold temperature underlies plant adaptation to different temperate and boreal climates. An extended chilling period is a signal for dormancy release of shoot apical and cambial meristems of many woody plants, herbaceous plant tubers and seeds of various plants as well as for the promotion of the floral transition in both monocarpic and polycarpic plant species (Cooke et al., 2012; Graeber et al., 2012; Romera-Branchat et al., 2014; Sonnewald and Sonnewald, 2014). Similarities and differences among these responses have been discussed a number of times (Chouard, 1960; Rohde and Bhalerao, 2007; Horvath, 2009). Here, we focus on the release of bud dormancy and the floral transition, post-embryonic responses of shoot meristems that occur in trees. A dormant meristem is unable to resume growth under favorable conditions; however, the dormant state is quantitative with endogenous and environmental signals continually altering the depth of dormancy (propensity for growth given advantageous conditions; Rohde and Bhalerao, 2007; Cooke et al., 2012). Studies in herbaceous plants have shown a similar quantitative relationship between length of the cold treatment and flowering time in subsequent inductive conditions (Wollenberg and Amasino, 2012). The environmental signals regulating the floral transition in trees are difficult to dissect due to a multi-year juvenile (non-flowering) phase and thus, large tree size at first flowering. However, the winter chilling period appears to be a signal for the seasonally recurring floral transition in adult *Populus* (Hsu et al., 2011).

The quiescence of cells during chilling-induced dormancy release has been suggested as a possible discrepancy between vernalization and dormancy release (Chouard, 1960; Rohde and Bhalerao, 2007). However, cell division appears to only be required to stabilize the epigenetic-mediated vernalization response in *Arabidopsis* (Finnegan and Dennis, 2007). Moreover, a stable versus transient vernalization response differentiates monocarpic *Arabidopsis* from its relative, polycarpic *Arabis alpina* (Wang et al., 2009). As has been recently reviewed (Rios et al., 2014), epigenetic states are altered in dormant versus non-dormant buds. Deletion within the locus containing tandemly repeated *DORMANCY ASSOCIATED MADS-BOX (DAM*) genes has been linked to the *evergrowing* peach locus that prevents dormancy (Bielenberg et al., 2008). *DAM6* is upregulated during dormancy and its repression during dormancy release correlates with changes in histone modifications (Jimenez et al., 2010; Yamane et al., 2011; Leida et al., 2012). Evolutionarily diverse herbaceous plants employ unrelated genes in the vernalization response, indicating that this signaling pathway has evolved independently multiple times (Andres and Coupland, 2012; Ream et al., 2012). However, the downregulation of a floral repressor by an extended chilling period is a shared feature and at least in some tree taxa and perennial herbaceous plants such as leafy spurge, *DAM* could have an analogous role in bud dormancy (Horvath et al., 2010; Jimenez et al., 2010; Sasaki et al., 2011). In addition, the vernalization pathways in different plants converge on related flowering time genes, in particular, the broadly conserved promoter of the floral transition, *FLOWERING LOCUS T* (*FT*; Kardailsky et al., 1999; Kobayashi et al., 1999). *FT* activity is countered by the related gene *TERMINAL FLOWER1* (*TFL1*) that maintains indeterminate meristems (Bradley et al., 1996; Ratcliffe et al., 1998). In this review, we discuss seasonal shoot development contexts and roles of *Populus* orthologs of *FT* and *TFL1*, focusing on what these genes indicate about regulatory overlap between dormancy release and vernalization. Approaches to extending our knowledge of the regulatory networks governing these processes are also discussed.

## WINTER TO SPRING SHOOT DEVELOPMENT IN *Populus*

**FIGURE 1:**
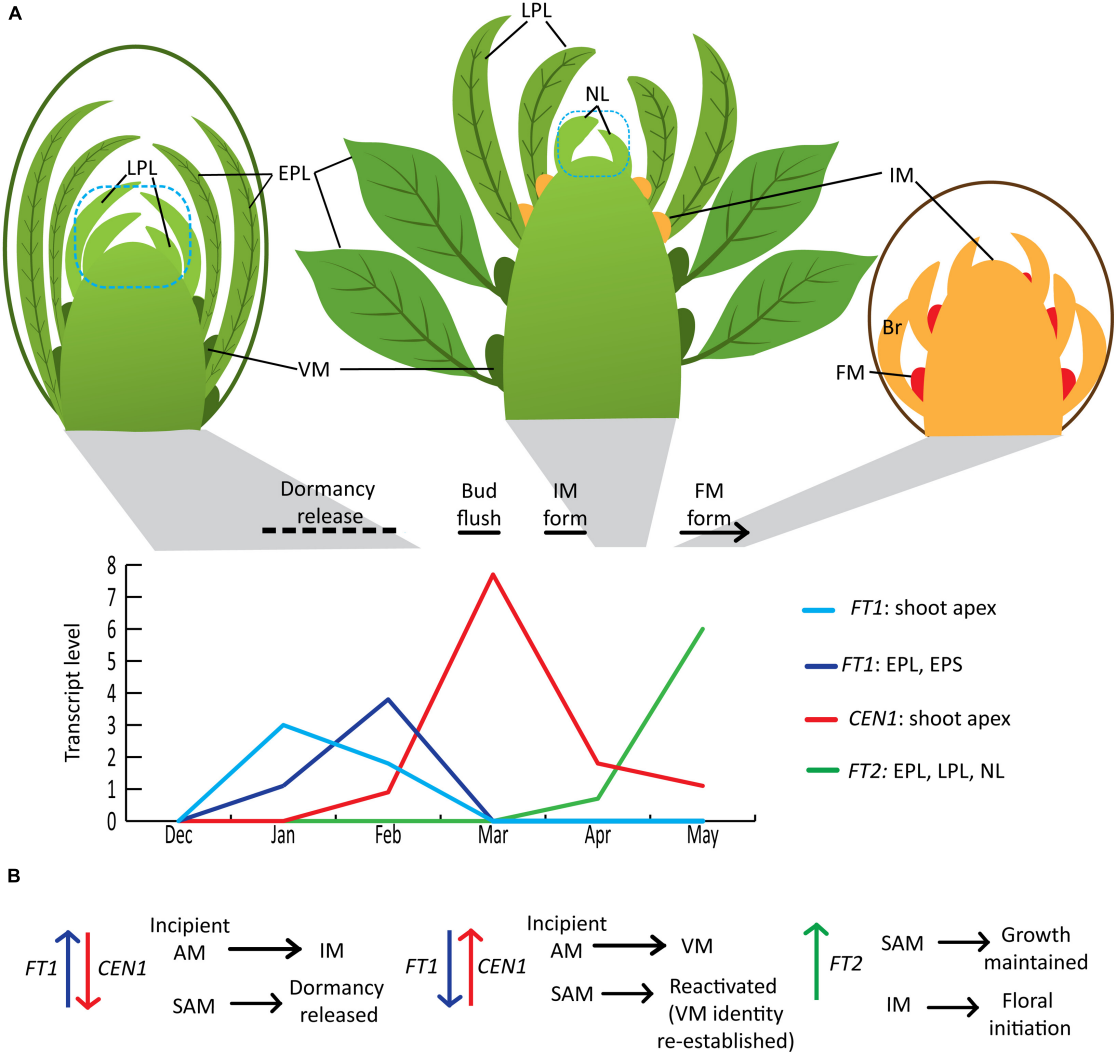
**Winter to spring shoot development and roles of *FT1*, *FT2*, and *CEN1* in *Populus*. (A)** Seasonal shoot phenology and *FT1*, *FT2,* and *CEN1* expression associated with dormancy release and the initiation of flowering. Seasonal phenology and relative gene expression patterns are based on study of adult *Populus deltoides* in Mississippi, USA (Yuceer et al., 2003; Hsu et al., 2011) with the exception of the timing of dormancy release, which is an estimate based on the apparent correlation between increasing *FT1* expression and dormancy release in controlled environment studies reported by Rinne et al. (2011). The blue dotted line indicates that the shoot apex sample used for expression studies included leaf primordia smaller than 1 mm (e.g., LPL in the dormant bud). Note that the diagrams are not to scale and the number of EPL in a bud can be several more than the depicted number. In addition, an inflorescence typically forms more than 100 flowers that develop within the inflorescence bud over the course of the growing season and after winter dormancy, anthesis occurs the following spring. **(B)** Conceptual model of how seasonal changes in levels of *FT1*, *CEN1,* and *FT2* sequentially contribute to the regulation of dormancy release, meristem identity and growth. AM, axillary meristem; IM, inflorescence meristem; FM, floral meristem; VM, vegetative meristem; EPL, early preformed leaves; EPS, early preformed shoot; LPL, late preformed leaves; NL, neoformed leaves; Br, bracts.

In *Populus*, both vegetative and floral shoot development proceeds acropetally. A dormant vegetative bud contains several preformed leaves that can be described as embryonic leaves/early preformed leaves (EPLs) or leaf primordia/late preformed leaves (LPLs) based on size, presence of trichomes and differentiation of blade and petiole (Critchfield, 1960; Yuceer et al., 2003; **Figure 1A**). In addition, meristematic domes have already formed in the axils of EPLs (Yuceer et al., 2003). The chilling sum needed to release dormancy varies with genotype, but dormancy is typically released several weeks prior to bud flush, whose timing is primarily determined by accumulated heat units (Cooke et al., 2012). Dormancy is released gradually and dormancy depth can be monitored by moving ramets at regular intervals to growth-promoting conditions and monitoring the time to bud flush or other features related to growth resumption. In *Populus*, apical meristems remain vegetative. In adult trees, some axillary meristems transition to inflorescence meristems that subsequently initiate bracts and then floral meristems in the bract axils (Brunner, 2010). Axillary inflorescence buds are microscopically distinguishable from axillary vegetative buds a few weeks after vegetative bud flush, when they begin to elongate and initiate bracts (Boes and Strauss, 1994; Yuceer et al., 2003). The development of the newly initiated inflorescence buds continues during the growing season and floral organ differentiation is mostly completed within the bud before winter dormancy. Inflorescence bud flush occurs before vegetative bud flush the following year. However, dormancy release of inflorescence buds has not been studied in *Populus*; thus, this review is limited to vegetative bud flush and the transition of some of the vegetative shoot’s axillary meristems to flowering. *Populus* exhibits an indeterminate growth pattern in that vegetative shoots will continue to elongate, initiating new leaves [neoformed leaves (NLs)] until the critical daylength for growth cessation occurs as long as other conditions are suitable for growth. However, as trees increase in size/age, the proportion of shoots that exhibit indeterminate growth decreases and a tree contains shoots ranging from determinate (short shoots with only preformed leaves) to fully indeterminate shoots (Critchfield, 1960). Moreover, inflorescence buds are most frequently present on determinate shoots or shoots that initiate only a few NLs (Yuceer et al., 2003; Brunner, 2010). A detailed seasonal time course study in *Populus deltoides* indicated that only meristems in the axils of LPLs can transition to flowering (i.e., convert to inflorescence meristems; Yuceer et al., 2003), whereas axillary meristems of EPL and NL are vegetative. Thus, in adult trees, there is a seasonal window where certain axillary meristems are able to transition to inflorescence meristems. The developmental state of the leaf and/or its axillary meristem could be factors in determining competency for the floral transition.

## *Populus FT/TFL1* FAMILY MEMBERS: FUNCTIONS AND ADAPTIVE VARIATION

The *Populus trichocarpa* genome contains six genes encoding full-length proteins belonging to the *CENTRORADIALIS (CEN)/TFL1/SELF-PRUNING (SP; CETS*) family (Pnueli et al., 2001; Mohamed et al., 2010). This includes two members (*FT1, FT2*) of the *FT* subclade and two members (*CEN1, CEN2*) of the *CEN/TFL1/SP* subclade. Driven by the 35S promoter, *FT1* is a strong promoter of precocious flowering in *Populus* and, in particular, induces the formation of wild-type inflorescences (Bohlenius et al., 2006). In contrast, *FT2* only induces the formation of individual flowers (Hsu et al., 2006). Subsequent studies of gene expression over a seasonal cycle in various organs/tissues showed that the two paralogs have distinct seasonal expression peaks and tissue expression patterns (Hsu et al., 2011; **Figure 1A**). Controlled environment studies showed that *FT1* is upregulated during exposure to chilling temperatures, whereas long days and warmer temperatures promote *FT2* expression, which promotes vegetative growth (Bohlenius et al., 2006; Hsu et al., 2011; Rinne et al., 2011). Similar to the aforementioned results concerning the floral transition, *FT1* can compensate for reduction in *FT2* to maintain growth under short-daylengths, but not to an equivalent degree (Bohlenius et al., 2006; Hsu et al., 2011). Thus, the *Populus FT1/FT2* paralogs are a striking example of gene duplication followed by a dramatic differentiation in regulation and a subtle differentiation in protein function. In *Arabidopsis* and other herbaceous plants, vernalization enables *FT* expression, but *FT* is not upregulated until the plant is exposed to floral inductive conditions (Andres and Coupland, 2012; Ream et al., 2012). In contrast, *FT1*’s upregulation during chilling suggests it could have a role in mediating the vernalization response. Given *FT2*’s expression in developing inflorescences and ability to induce single flowers when overexpressed, *FT2* could have a role in floral meristem initiation within the developing inflorescence shoot (Hsu et al., 2006, 2011).

The first population genomics study in *P. trichocarpa* has further shown the importance of *FT1* and *FT2* in seasonal phenology (Evans et al., 2014). With collections spanning much of the natural range of *P. trichocarpa,* naturally segregating variation in *FT2* was associated with time of fall bud set in accordance with previous functional studies. Conversely, *FT1* variation was associated with time of spring bud flush. In both cases, despite a number of non-synonymous single nucleotide polymorphisms (SNPs) within the coding regions of the paralogs, the strongest signals of association were intronic, suggesting that regulatory variation, rather than protein changes, drive phenological adaptation. Furthermore, patterns of polymorphism surrounding both paralogs are consistent with strong positive and divergent selection related to climatic variables across the species’ range, just as quantitative genetic patterns of spring bud flush and fall bud set display climatic correlations themselves (Howe et al., 2003; Evans et al., 2014). Differences in bud flush phenology can be due to differences in dormancy release phenology as well as differences in post-dormancy heat sum requirements for bud flush. However, there is indirect evidence that *FT1* could promote dormancy release. First, in controlled environment studies, *FT1* upregulation and dormancy release appeared to be correlated (Rinne et al., 2011), though additional studies are needed to determine if *FT1* upregulation is indeed a marker for dormancy release in *Populus*. The second line of evidence comes from studies of *CEN1* in *Populus* and the antagonistic functions of *FT* and *TFL1* in *Arabidopsis* and other plants (discussed in the next section). *CEN1* is dramatically upregulated in the shoot apex around the time of bud flush (Mohamed et al., 2010; Rinne et al., 2011) yet *35S::CEN1* transgenics showed markedly delayed bud flush under field conditions (Mohamed et al., 2010). Moreover, controlled environment studies showed that dormancy release is delayed in the *35S::CEN1* transgenics and accelerated in transgenics with *CEN1* and *CEN2* downregulated. *CEN1/CEN2* downregulation also resulted in an earlier onset of first flowering and more intense flowering (i.e., more axillary meristems converted to inflorescence meristems) under field conditions. Considered together, these results suggest that relative levels of *FT1* and *CEN1* could contribute to dormancy release and meristem identity (**Figure 1B**). In this model, a high *FT1* level relative to *CEN1* promotes dormancy release as well as the transition of incipient axillary meristems to inflorescence meristems. As the season progresses, decreasing *FT1* and rapidly increasing *CEN1* ensures that newly initiating axillary meristems are vegetative and also reactivates or re-establishes the vegetative identity of shoot apical meristems. After bud flush, as *CEN1* expression declines, *FT2* expression increases, supporting that *FT2*’s main role is to maintain growth rather than initiate it. Although functions of *CEN1* and *CEN2* have not been separated, *CEN2* expression is very low in all the samples shown in **Figure 1B**, suggesting that *CEN1* is the paralog involved in dormancy release and repressing the floral transition. However, *CEN2* is expressed in the developing inflorescence and could have a role in maintaining the inflorescence meristem (Igasaki et al., 2008; Mohamed et al., 2010). Delineation of *CEN1* versus *CEN2* functions and validating *FT1*’s proposed function in dormancy release as well as *FT2’*s possible role in floral initiation/commitment requires paralog-specific downregulation. This was not achieved with previously studied RNAi transgenics, but perhaps can be accomplished using artificial microRNAs or the CRISPR/Cas9 system.

## INTERCELLULAR CONNECTIONS AND SPATIOTEMPORAL CONTEXTS OF *Populus FT1/2* AND *CEN1* ACTIVITY

A central feature of the dormant bud is that symplastic conduits are blocked by callose deposition in plasmodesmata (PD) and these are opened during chilling-induced dormancy release (Rinne et al., 2001, 2011). Whereas *CEN1* and *FT2* expression peaks are clearly subsequent to dormancy release, *FT1* upregulation overlapped with the reopening of PD in controlled environment studies of juvenile trees (Rinne et al., 2011). Dormancy release cannot be directly assessed in the field, and the relationship between *FT1* expression and the open/closed status of PD in adult, flowering trees is uncertain. All of a tree’s buds are not released from dormancy at the same time and the requirements and timing of dormancy transitions might change as a tree ages (Rohde and Bhalerao, 2007; Cooke et al., 2012). Similarly, there is evidence that within the bud, EPL are released from dormancy before the SAM (Rinne et al., 2011). Glycosyl hydrolase family 17 (GH17) proteins (1,3-β-glucanases) degrade callose and the upregulation and localization of some *Populus* GH17 family members correlates with dormancy release/opening of PD (Rinne et al., 2011). Intriguingly, constitutive and heat-inducible expression of *FT1* in transgenic *Populus* upregulated a *GH17* gene and altered the expression of a large number of genes involved in carbohydrate, protein and lipid metabolism (Hsu et al., 2011) that would be expected to occur with dormancy release (Druart et al., 2007; Rohde et al., 2007). Although the *GH17* gene (Potri.004G153800) was not among those studied in relation to dormancy by Rinne et al. (2011), this suggests a possible mechanism for *FT1*-mediated dormancy release as well as that FT1 could have a role in controlling its own intercellular movement by promoting open PD. PD are dynamically regulated to control various developmental processes (Brunkard et al., 2013; Stahl and Simon, 2013; Paul et al., 2014; Sager and Lee, 2014). In particular, PD trafficking within the SAM is altered during the floral transition in *Arabidopsis*. Although the importance of FT and TFL1 intercellular movement to flowering is well known (Conti and Bradley, 2007; Corbesier et al., 2007; Jaeger and Wigge, 2007), to our knowledge, the possibility that they have a role in regulating PD trafficking has not been studied. There are also details of *FT1* expression within the bud that still need to be determined, specifically in regards to the shoot apex (**Figure 1A**). Hsu et al. (2011) showed that *FT1* is expressed in the shoot apex as well as the EPLs and embryonic stem, but the shoot apex sample encompassed the SAM, rib zone as well as the youngest leaf primordia (i.e., LPL in the bud) and their axils where inflorescence meristems may develop. Also, *FT1* expression in the shoot apex peaks earlier than in the EPL in adult *P. deltoides* (Hsu et al., 2011; **Figure 1A**). This could potentially have a role in differentiating *FT1* functions. A number of scenarios are possible; for example, *FT1* expression in the LPL axillary mersitems could promote the floral transition, but dormancy release of the SAM depend on import of the FT1 protein from EPL. Thus, filling in these details could be important to understanding the proposed dual roles of *FT1* in the floral transition and dormancy release in adult trees as well as how only dormancy release and not flowering is promoted in juvenile trees.

Accumulating evidence supports that the local balance of FT/TFL1 controls growth and flowering, but the spatiotemporal elaboration of these balances, including both gene expression and transport patterns, differ (Shalit et al., 2009; Jaeger et al., 2013). Hence, aspects of growth and flowering patterns differ among plants. In *Arabidopsis*, both *FT* and *TFL1* expression levels rise after floral induction with high TFL1 levels in the center of the shoot apex effectively counterbalancing FT to maintain an indeterminate apical inflorescence meristem (Jaeger et al., 2013). Following the transition to flowering in tomato, apical meristems initiate a number of leaves and then terminate in a flower. The tomato *FT* ortholog *SINGLE FLOWER TRUSS* (*SFT)* and *SP* regulate not only the sympodial growth and flowering pattern but also rate of leaf maturation and compound leaf complexity (Shalit et al., 2009). Both in the primary shoot and the sympodial shoots, *SFT* and *SP* show opposite gradients of expression in a leaf developmental gradient. *SP* is high in young leaves and *SFT* is high in older leaves. Thus, the roles of *FT1/FT2* and *CEN1/CEN2* in dormancy release, growth and flowering could be viewed as a variation on a common theme of local FT/TFL1 balance determining multiple aspects of growth and flowering. In the case of *Populus*, *FT* functions and likely *TFL1* functions have been parsed out among paralogs. Thousands of gene pairs are retained from the Salicoid whole genome duplication event (Tuskan et al., 2006), which remains a key feature influencing how selection shapes the genome (Evans et al., 2014), but also provides the possibility of altered, specialized, or novel function of duplicate pairs.

## EXTENDING THE VERNALIZATION AND CHILLING-INDUCED DORMANCY RELEASE NETWORKS

Regulation of local FT/TFL1 balance may be a broadly shared component of dormancy release and vernalization pathways. Nonetheless, the upstream networks regulating this balance as well as downstream genes need to be identified to fully address the question of the degree of overlap among vernalization and dormancy release networks. By analogy to the distinct vernalization response genes in wheat, beet and *Arabidopsis* (Ream et al., 2012), other genes involved in dormancy release could vary among diverse woody plant taxa. However, the role of *DAM* genes in promoting dormancy appears to be conserved among different Fabidae taxa, including *Populus* (Bielenberg et al., 2008; Horvath et al., 2010; Sasaki et al., 2011). Although Japanese pear DAM did not alter pear *FT* promoter-driven luciferase expression in tobacco leaves (Saito et al., 2014), further studies are needed to determine if *DAM* and *FT* are part of the same regulatory pathway or act independently. Study of natural variation in flowering time has clearly helped develop a mechanistic understanding of the vernalization response pathway in *Arabidopsis* (Amasino, 2010). Population genomics studies are likely essential to revealing the mechanisms underpinning seasonal development in *Populus* and other trees, where generation of extensive, tagged loss-of-function mutant populations is not feasible and the developmental processes take many weeks to study. However, bridging the gap between genetic associations and endogenous function is a major challenge. Through genome-wide association mapping and selection scan in 544 *P. trichocarpa* trees from a wide range of latitudes, Evans et al. (2014) have provided the first unbiased identification of genes associated with adaptation in tree bud phenology, which included *FT1/2*. Correspondingly, we have focused on *FT1/2* and antagonists *CEN1/2* because they are the exceptions—genes for which direct functional analysis via transgenic *Populus* mutants supports the genetic association findings. However, most of the genes identified by Evans et al. (2014) were of unknown function, at least above the level of basic molecular/cellular categorization, or not known to influence phenology. Combining association studies with genomic scans of spatial variation, positive selection, and duplicated genes within species (e.g., *FT1/2*), as well as with comparative analyses across species, will provide clues to the shared and divergent pathways of dormancy and vernalization. However, by identifying possible variants for further functional study, this will only begin to leverage the potential of population genomics.

We suggest that identifying molecular networks of dormancy release and growth resumption that capture the spatiotemporal complexity of these seasonal developmental processes and some of the diversity could be a major step toward connecting adaptive variation to endogenous function. Most of the SNPs associated with bud phenology were located in non-coding regions (Evans et al., 2014), suggesting that the regulation of gene expression, the transcription of non-coding RNAs, and alternate splicing are important for adaptation to different climates. All of these can be revealed by next-generation transcriptomics, especially when a high quality reference genome is available as is the case for *P. trichocarpa*. For such an approach to provide meaningful mechanistic and functional inference, multiple tissues and time points need to be studied and controlled environment studies can best connect dormancy stages to specific environmental variables and transcriptomic changes. This approach would preclude study of large numbers of genotypes to match the number used for population genomics analyses. Nonetheless, several distinct latitudinal or elevational ecotypes could be studied by such an approach to reveal some of the underlying transcriptional and post-transcriptional differences underpinning adaptive variation. This variation and the resulting functional transcriptomic networks could be integrated with GWAS, genetical genomics, and comparative genomics approaches to identify regulatory elements. This could also serve as the basis for functional characterization of key nodes via transgenic manipulation of *Populus* and for identifying protein–protein interaction networks around them. Similar mechanistic frameworks can be developed in different tree taxa to reveal the degree of conservation in dormancy release pathways. While the length of time to reach sexual maturity in *Populus* creates difficulties, its sister group, *Salix,* is an excellent candidate for transcriptomic studies of the floral transition due to its much shorter generation time and smaller stature. Moreover, genetic mapping in *Salix* has shown the genomes to be largely syntenic (Berlin et al., 2010) and the *Salix purpurea* genome sequence is available (DOE-JGI, 2014).

## Conflict of Interest Statement

The authors declare that the research was conducted in the absence of any commercial or financial relationships that could be construed as a potential conflict of interest.
